# Reversible Thyroid Nodule in Van Wyk–Grumbach Syndrome: An Unusual Case Report

**DOI:** 10.14740/jmc5386

**Published:** 2026-10-02

**Authors:** Laila A. AlBishi

**Affiliations:** Department of Pediatrics, Faculty of Medicine, University of Tabuk, PO Box 71491, Tabuk, Saudi Arabia. Email: lalbishi@ut.edu.sa

**Keywords:** Van Wyk–Grumbach syndrome, Thyroid nodule, Juvenile hypothyroidism, Precocious puberty, Autoimmune thyroiditis

## Abstract

Van Wyk–Grumbach syndrome (VWGS) is a rare complication of longstanding, untreated primary hypothyroidism in children. It is characterized by isosexual precocious puberty, delayed bone age, and multicystic ovarian enlargement despite the presence of severe hypothyroidism. Although thyroid enlargement is common, a discrete thyroid nodule has not previously been documented in this syndrome. This study presents a 6-year-old Saudi girl who exhibited a 2-day history of vaginal hemorrhage and a history of breast development for 6 months. The physical examination demonstrated a palpable right thyroid nodule. The laboratory evaluation revealed a severe primary hypothyroidism, high thyroid autoantibodies, elevated prolactin levels, and prepubertal levels of basal gonadotropins. A thyroid ultrasound demonstrated a well-defined, hypoechoic nodule. Further testing revealed that the child had a delayed bone age, multi-cystic ovaries, and pituitary hyperplasia. After levothyroxine treatment, the patient’s thyroid hormone levels normalized. In addition, there was progressive shrinkage of the thyroid nodule, pituitary hypertrophy, and ovarian cysts, along with significant improvement in linear growth. This case adds to the previously unreported association of a reversible thyroid nodule with VWGS and highlights the importance of early diagnosis and appropriate management of severe hypothyroidism to prevent unnecessary or invasive diagnostic tests.

## Introduction

Van Wyk–Grumbach syndrome (VWGS), first described in 1960, is a rare clinical manifestation of longstanding juvenile hypothyroidism characterized by the paradoxical occurrence of isosexual precocious puberty despite severe thyroid hormone deficiency [1]. The syndrome classically manifests breast development and vaginal bleeding in girls or testicular enlargement without virilization in boys, accompanied by delayed bone age and multi-cystic gonadal enlargement [2]. These manifestations result from chronic elevation of thyroid-stimulating hormone (TSH), which exhibits structural homology with follicle-stimulating hormone (FSH) and can activate FSH receptors at supraphysiologic concentrations, leading to gonadal hyperstimulation [3]. Concomitant hyperprolactinemia, resulting from unopposed thyrotropin-releasing hormone (TRH) stimulation of lactotrophs, further enhances ovarian sensitivity to gonadotropins and contributes to the clinical phenotype [4].

Although VWGS has been well-characterized with respect to its reproductive and systemic manifestations, focal structural changes within the thyroid gland, specifically, discrete nodules, have received limited attention. Most reports focus on diffuse thyroid enlargement (goiter) as a consequence of chronic TSH stimulation, consistent with the pathophysiology of autoimmune thyroiditis or iodine deficiency [5, 6]. However, there are no prior reports describing discrete thyroid nodules as a clinical manifestation of VWGS. In this context, it is particularly pertinent that elevated TSH levels over extended periods may promote the growth of thyroid tissue and ultimately lead to follicular hyperplasia and nodule formation in various clinical contexts [7]. The present case report describes a 6-year-old girl with VWGS who presented with a palpable thyroid nodule in addition to the classic features of the syndrome. To our knowledge, no prior case of VWGS has reported a discrete, reversible thyroid nodule. This is an important observation as it suggests that long-term high levels of TSH may cause localized structural changes in the thyroid in severely hypothyroid children. It also emphasizes the importance of conducting a comprehensive structural evaluation of the thyroid by physically examining the child and using ultrasound when children present with VWGS.

This case also features the broader clinical significance of recognizing VWGS as a reversible cause of precocious puberty and pituitary enlargement. Misdiagnosis may result in unnecessary invasive investigations, including pituitary surgery, and inappropriate hormonal interventions [8]. Early recognition and treatment with levothyroxine leads to gradual but complete resolution of all endocrine abnormalities, including regression of ovarian cysts, pituitary hyperplasia [9], and possibly regression of a discrete thyroid nodule.

## Case Report

### Investigations

A 6-year-old girl from Saudi Arabia came to the pediatric endocrine clinic with a 2-day history of vaginal bleeding. Her parents reported that she had breast enlargement on both sides over the previous 6 months, with no growth underarm or pubic hair. They also noticed that she had become less social and demonstrated decreased concentration at her school. These variations were corroborated by the schoolteacher. A full-body checkup revealed no issues, including headaches, changes in vision, unusual movements, or behavioral shifts. There was no history of local trauma, abnormal bleeding from other sites, dysuria, or foreign-object insertion.

Two years before this child’s presentation, the patient had been assessed for poor appetite but never returned for follow-up. At that time, her height was 97 cm, her weight was 16 kg (10th and 25th centiles, respectively), and she had no abnormal findings recorded. The patient’s family history was significant for consanguinity, as well as her mother’s history of maternal autoimmune thyroiditis, for which she is currently on treatment. The patient is the second of her family’s children delivered vaginally at term, with an uneventful outcome. The child had met all developmental milestones and was up to date on vaccinations.

Physical examination of the patient revealed a dull-looking face with puffiness around both eyes and cheek fullness. Figure 1 displays growth measurement data for this patient. Her height of 101.3 cm is below the third percentile, corresponding to a −2.5 standard deviation score (SDS), while her weight of 26 kg places her at the 90th percentile for her age, and her BMI is 25.49 kg/m^2^. Her mother’s and father’s heights were 152.4 and 158.2 cm, respectively; her calculated target height is 148.8 cm. Assessment for puberty revealed a girl in Tanner stage B3P1 (B for breast development; P for pubic hair), but no axillary hair, no virilization, and no galactorrhea. On neck examination, there was a palpable right thyroid mass approximately 1.2 × 1.5 × 1.0 cm in size. It was smooth, firm in consistency, and non-tender. There was no cervical lymphadenopathy. The physical findings were consistent with a benign condition, but could not be ruled out for cancer by physical exam alone (a further ultrasound demonstrated more on the thyroid nodule). Further signs were negative for café au lait spots, bone deformities, and papilledema.

**Figure 1:**
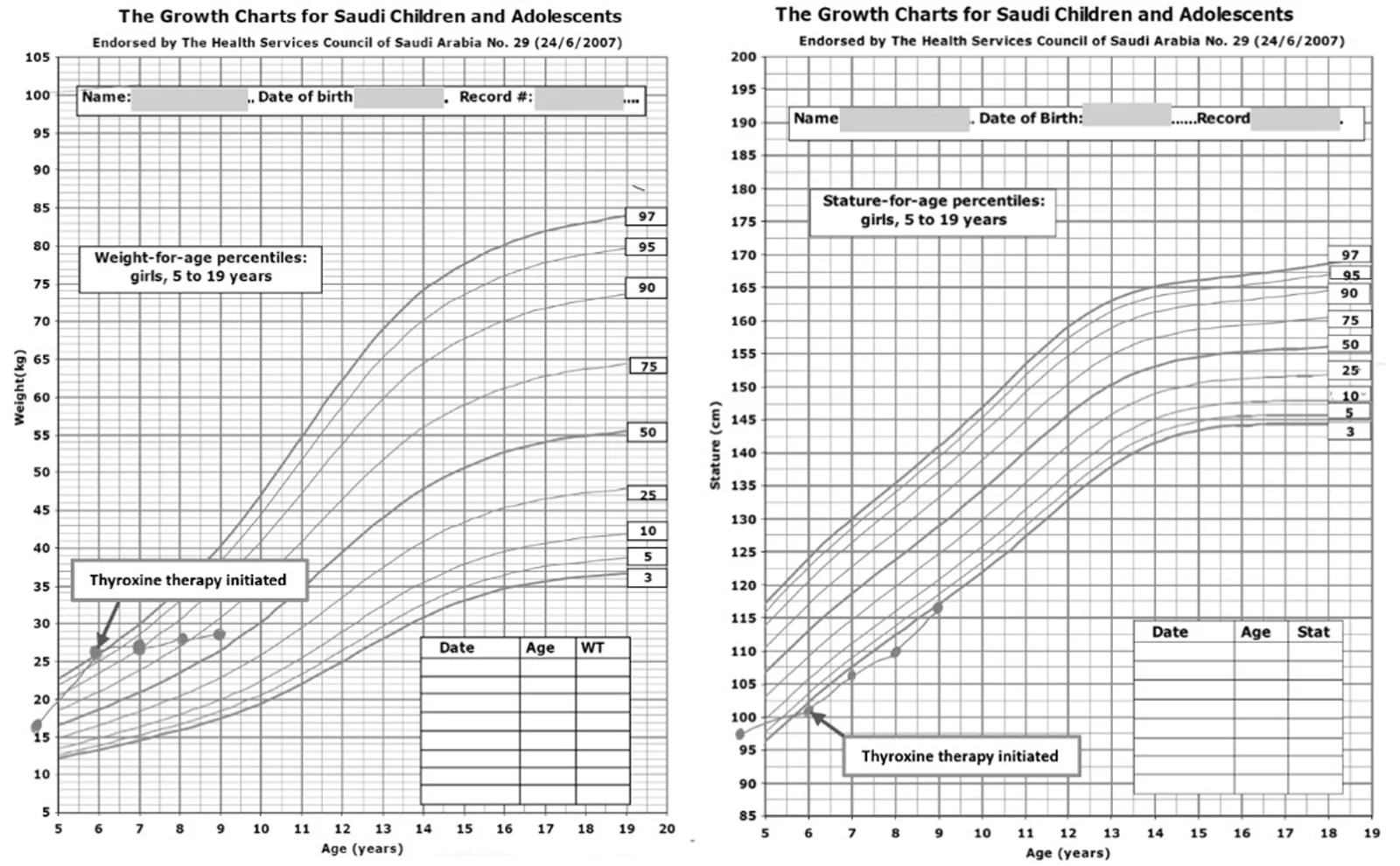
Anthropometric measurements before and after levothyroxine therapy. Weight-for-age and height-for-age percentile charts for Saudi girls illustrating the patient’s growth trajectory at presentation. The plotted points indicate improvements in growth parameters before and after the initiation of levothyroxine therapy (arrow).

### Diagnosis

Laboratory results demonstrated that there was significant primary hypothyroidism based on very high TSH (> 75 mIU/L), low free thyroxine (FT4, 9.1 pmol/L), and low free triiodothyronine (FT3, 3.4 pmol/L). The lab data indicated significant glandular failure over time. The presence of high autoantibody activity confirmed autoimmune thyroiditis.

The prolactin (PRL) level was very high at 173 ng/mL and therefore consistent with TRH stimulating secretion. Gonadal hormone levels were prepubertal with luteinizing hormone (LH) of < 0.21 IU/L and follicle-stimulating hormone (FSH) of 7.01 U/L. Estradiol levels were higher than expected (82 pg/mL), suggesting ovarian hyperstimulation. Both progesterone and dehydroepiandrosterone sulfate (DHEA-S) were within the normal limits. This makes precocious puberty due to an adrenal gland abnormality unlikely.

Macrocytic hyperchromic anemia was identified via complete blood count (CBC). Anemia in association with hypothyroidism can be seen in approximately 55% of patients (with no identifiable nutritional deficits) [10]. Levels of insulin-like growth factor-1 (IGF-1) and insulin-like growth factor-binding protein-3 (IGFBP-3) were determined to be in the normal range for screening of short stature. All laboratory testing results, including thyroid function tests, autoantibodies, and CBC, are listed in Table 1. A thyroid ultrasound confirmed diffusely enlarged thyroid lobes with heterogeneous hypoechoic echotexture and increased vascularity, consistent with autoimmune thyroiditis. In the right thyroid lobe, there was a nodule measuring 1.2 × 1.5 × 1.0 cm (estimated volume 0.94 mL) with well-defined margins and no microcalcifications, no spread beyond the thyroid, and no significant vascularity. Accordingly, it was rated as American College of Radiology Thyroid Imaging Reporting and Data System category 3 (ACR TI-RADS 3) (mildly suspicious with a low chance of cancer) (Fig. 2a, b). A bone age X-ray, read using the Greulich-Pyle method, was 4.5 years, reported as delayed for the chronological age. This moderate delay suggests that the patient still has good potential for linear growth once thyroid hormone treatment is started. Pelvic ultrasound revealed bilateral multicystic ovaries. The right ovary measured 2.3 × 3.6 cm, and the left ovary measured 2.7 × 3.2 cm indicating a bilateral ovarian enlargement. Multiple peripheral follicles created a characteristic “necklace” appearance, reflecting chronic FSH stimulation in the absence of ovulation. The uterus was prepubertal in size and configuration (Fig. 2c, d). Pituitary magnetic resonance imaging (MRI) demonstrated a homogeneously enhancing sellar mass measuring 20 × 15 × 13 mm with extension into the suprasellar cistern and grade 1 cavernous sinus extension (Knosp classification), without optic chiasm compression. The pituitary stalk was midline with normal enhancement, and the posterior pituitary bright spot was preserved. These findings were consistent with pituitary hyperplasia secondary to chronic hypothyroidism rather than a true adenoma (Fig. 3a, b).

**Table 1:** Baseline and Longitudinal Follow-Up Laboratory Parameters in Patients Diagnosed With Van Wyk–Grumbach Syndrome

Parameter	At diagnosis	3 months	6 months	9 months	Reference range	Interpretation
Hematology						
Hemoglobin (g/dL)	10.6	11.8	12.2	12.4	11.5–14.5	Macrocytic hyperchromic anemia at baseline, normalized with treatment.
MCV (fL)	95.4	84.2	82.1	81.5	75–87	Elevated at baseline, normalized
MCH (pg)	30.9	27.8	26.9	26.2	24–30	
MCHC (g/dL)	32.3	33.1	32.8	32.6	31–35	Normal throughout
RDW (%)	13.7	13.2	12.9	12.8	11.6–14	
Thyroid function						
TSH (mIU/L)	> 75	4.2	2.1	1.8	0.25–5	Markedly elevated at baseline, normalized by 3 months.
FT4 (pmol/L)	9.1	18.3	17.1	16.8	13.9–22.1	Low at baseline, normalized by 3 months
FT3 (pmol/L)	3.4	5.2	5.0	4.9	3.8–8.6	
Thyroid autoantibodies						
Anti-TG (U/mL)	161.7	-	-	115.2	< 15	Positive, gradually declining
Anti-TPO (U/mL)	190.1	-	-	138.7	< 15	
Reproductive hormones						
LH (IU/L)	< 0.21	< 0.21	0.32	0.45	1.3–1.4	Prepubertal at baseline, gradually increasing
FSH (U/L)	7.01	3.21	2.45	2.12	1.9–2.3	Elevated at baseline, normalized
Prolactin (ng/mL)	173	28	18	14	4.0–25	Markedly elevated at baseline, normalized by 3 months
Estradiol (pg/mL)	82	35	22	18	Up to 27	Elevated at baseline, normalized
Progesterone (ng/mL)	0.04	0.05	0.06	0.05	< 0.35	Normal throughout
DHEA-S (µg/dL)	10.3	12.1	14.5	16.8	54.4–254.0	Low, gradually increasing with age
Growth Factors						
IGF-1 (ng/mL)	98	112	128	145	88–246	Normal, increasing with treatment
IGFBP-3 (µg/mL)	2.8	3.2	3.5	3.8	2.1–5.2	

Anti-TG: anti-thyroglobulin antibodies; Anti-TPO: anti-thyroid peroxidase antibodies; DHEA-S: dehydroepiandrosterone sulfate; FSH; follicle-stimulating hormone; FT4: free thyroxine; FT3: free triiodothyronine; IGF-1: insulin-like growth factor-1; IGFBP-3: insulin-like growth factor-binding protein-3; LH: luteinizing hormone; MCH: mean corpuscular hemoglobin; MCHC: mean corpuscular hemoglobin concentration; MCV: mean corpuscular volume; RDW: red cell distribution width; TSH: thyroid-stimulating hormone.

**Figure 2:**
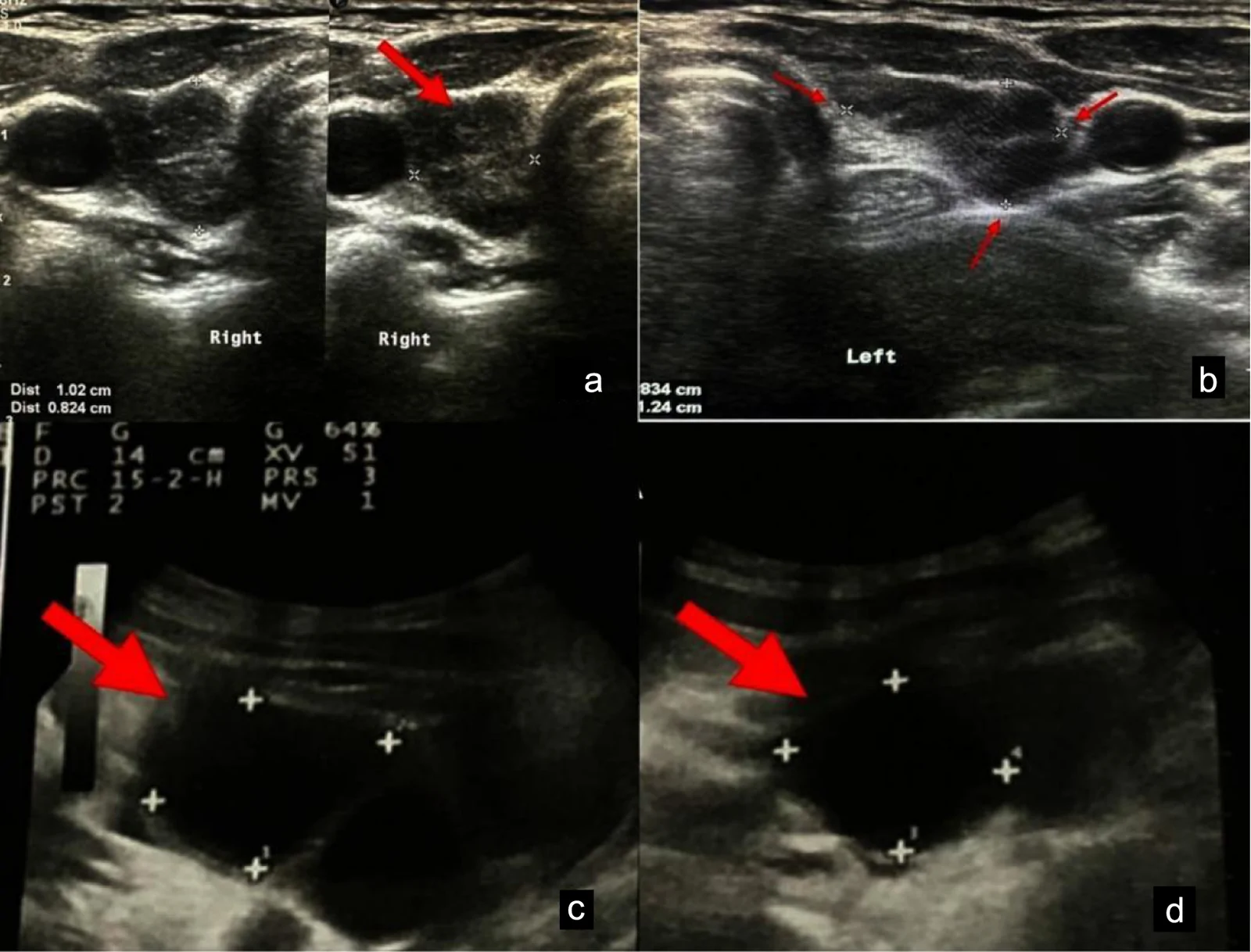
Thyroid and pelvic ultrasound imaging. (a, b) Thyroid ultrasound demonstrates a hypoechoic nodule in the right lobe. (c, d) Pelvic ultrasound showing bilateral multi-cystic ovaries (arrows) consistent with Van Wyk-Grumbach syndrome and a prepubertal uterus in size and configuration.

**Figure 3:**
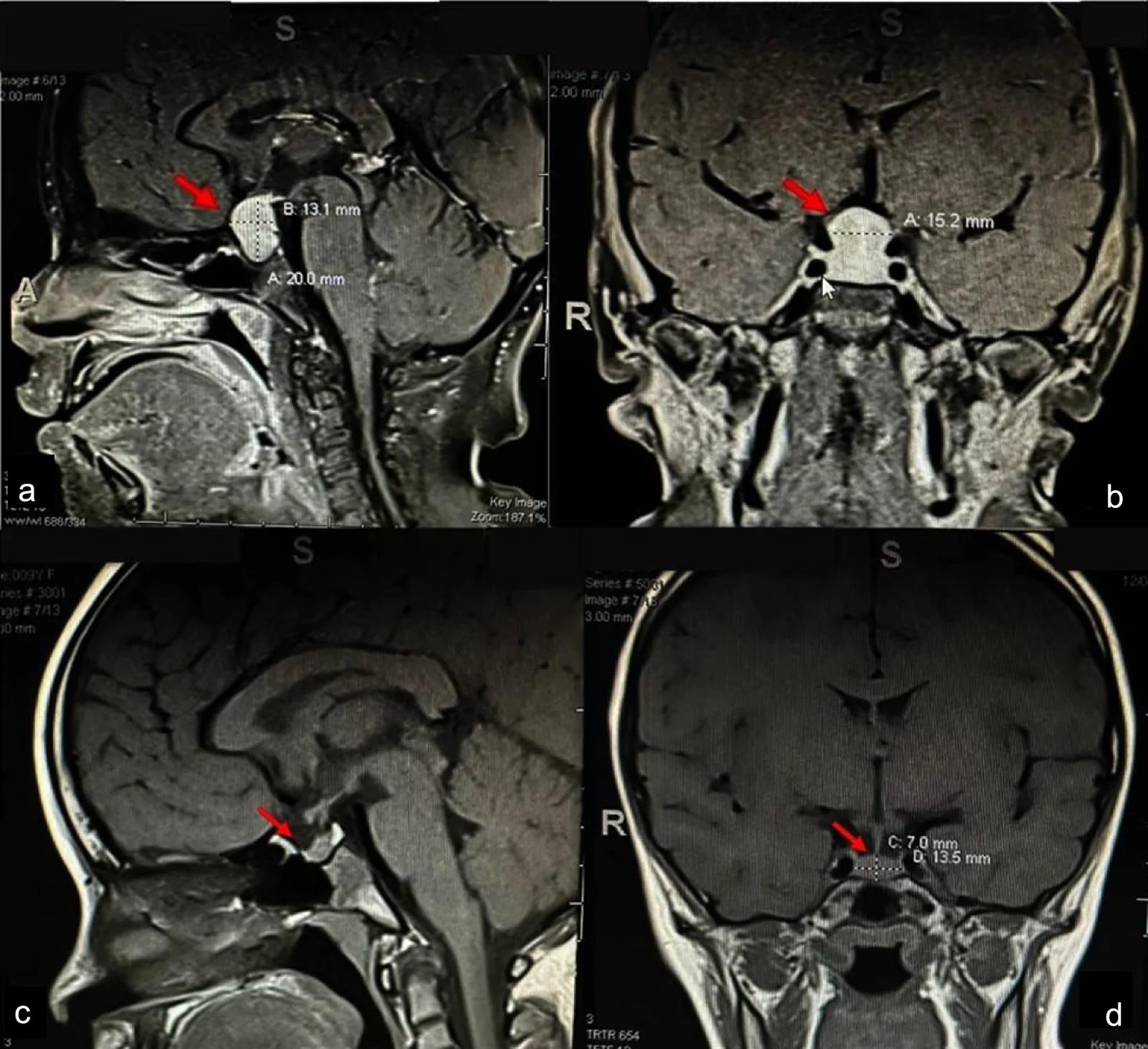
Pituitary magnetic resonance imaging. (a, b) Baseline sagittal and coronal T1-weighted post-contrast images showing pituitary hyperplasia with suprasellar extension (arrows). (c, d) Follow-up imaging at 9 months demonstrated a marked reduction in pituitary size after levothyroxine therapy.

### Treatment

The patient was initiated on levothyroxine replacement therapy at 100 µg/day, with thyroid function tests monitored and the dose titrated accordingly. The dose was selected based on pediatric guidelines for severe hypothyroidism, which recommend higher initial doses to achieve more rapid normalization of thyroid function. Monthly thyroid function tests were monitored to guide dose titration, with adjustments made to maintain TSH within the target range of 0.5–2.0 mIU/L to optimize regression of the thyroid nodule and pituitary hyperplasia [11]. No additional hormonal interventions were administered. Specifically, gonadotropin-releasing hormone (GnRH) agonists were not indicated, as the precocious puberty in VWGS is peripheral (gonadotropin-independent) and resolves with thyroid hormone replacement alone. Similarly, no surgical intervention was pursued for the pituitary mass or thyroid nodule, as both were expected to undergo progressive regression with normalization of thyroid function.

### Follow-up and outcomes

Vaginal bleeding resolved completely within 1 week of initiating levothyroxine therapy, likely reflecting withdrawal of estrogen support and endometrial shedding. This rapid response provided early reassurance to both the patient and her family. During 3 months of follow-up, thyroid function tests normalized: TSH decreased from > 75 to 4.2 mIU/L and FT4 increased from 9.1 to 18.3 pmol/L (Fig. 4; Table 1). Prolactin levels also dropped, and TSH normalized to 28 ng/mL by month 3. This trend confirmed that hyperprolactinemia had arisen from chronic TRH overstimulation.

**Figure 4:**
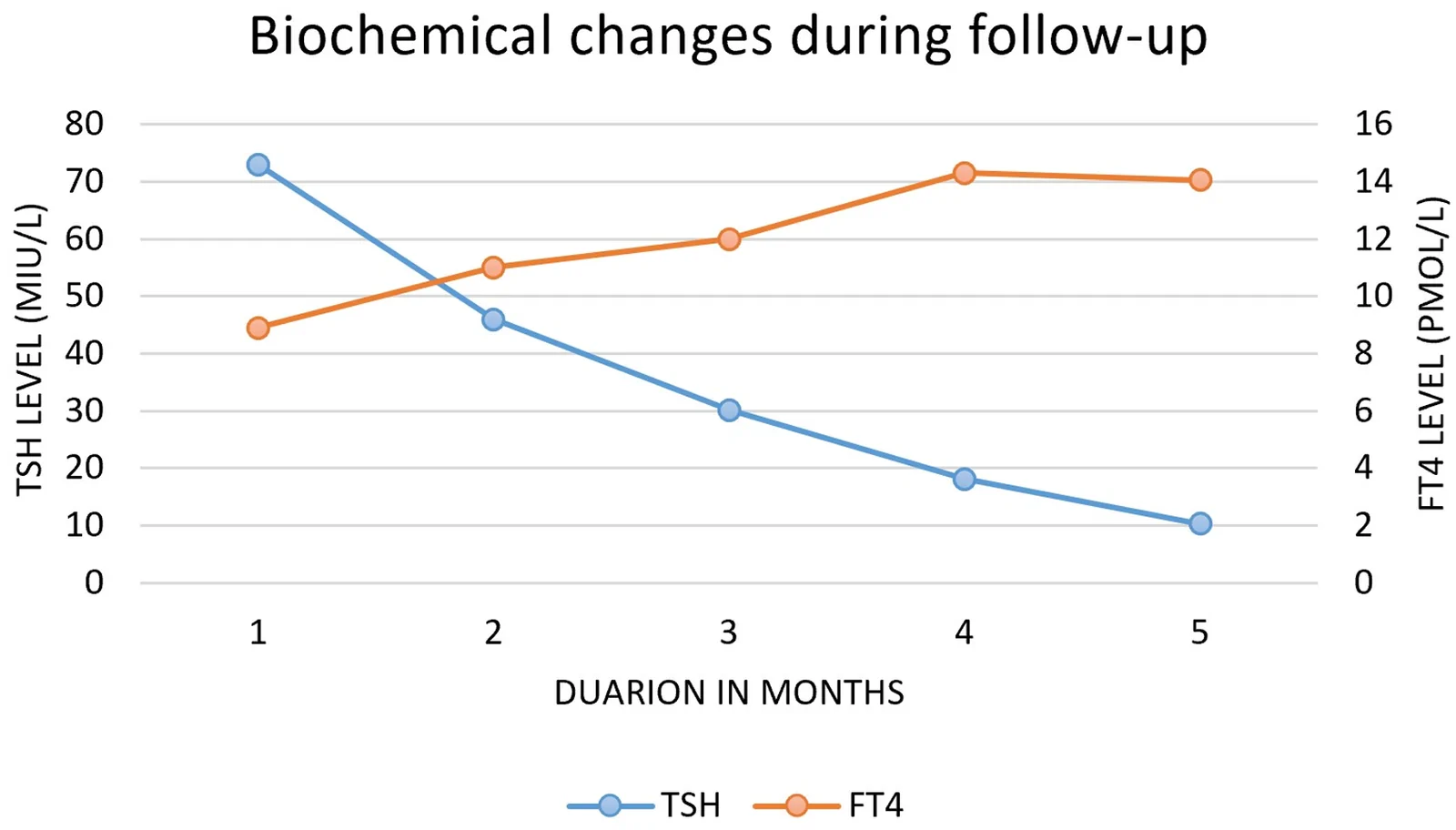
Temporal changes in thyroid function tests during levothyroxine therapy. Serial measurements of thyroid-stimulating hormone (TSH) and free thyroxine (FT4) during follow-up after initiation of levothyroxine therapy. The graph demonstrates progressive normalization of thyroid function, with a marked decline in TSH levels accompanied by a corresponding increase in FT4 concentrations over the follow-up period.

Follow-up thyroid ultrasounds showed a steady decrease in the size of the thyroid nodule. After 6 months of levothyroxine therapy, the nodule had shrunk to 0.7 × 0.9 × 0.6 cm (volume 0.20 mL), representing a 79% reduction in volume. The close temporal and quantitative correlation between TSH reduction and nodule volume regression provides strong support for a TSH-driven, reversible mechanism rather than a true neoplastic process.

Follow-up pelvic ultrasound at 9 months showed complete resolution of the previously noted multicystic ovarian changes, with normalization of ovarian volumes and return to normal prepubertal appearance of less than 2 cm. Repeat pituitary magnetic resonance imaging (MRI) at 6 months demonstrated a marked reduction in pituitary size from 20 × 15 × 13 to 12 × 10 × 8 mm (volume 0.5 mL; 75% reduction), with resolution of suprasellar extension and restoration of normal pituitary configuration (Fig. 3c, d). This dramatic regression confirmed the diagnosis of reversible pituitary hyperplasia rather than a true adenoma.

Anthropometric parameters improved over the follow-up period. At 9 months, the patient’s height was 106.8 cm (−2.0 SDS), representing a gain of 5.5 cm, indicating substantial catch-up growth (the normal growth velocity for a 6-year-old child is approximately 5–6 cm per year). Weight remained stable at 26.5 kg. Breast development showed gradual regression, with Tanner stage decreasing from B3 to B2 by 9 months. No further episodes of vaginal bleeding occurred. The patient’s school performance and social engagement improved markedly, as reported by parents and teachers.

At the most recent follow-up (12 months post-treatment initiation), the patient remains on levothyroxine 50 µg daily. Thyroid function is stable (TSH 1.8 mIU/L, FT4 16.2 pmol/L). Long-term monitoring is planned to assess final adult height, pubertal progression, and thyroid nodule status.

## Discussion

This case documents the first report of a discrete reversible thyroid nodule with VWGS, thereby expanding the recognized clinical spectrum of this rare disorder. The patient presented all classic features of VWGS including severe hypothyroidism, isosexual precocious puberty, multi-cystic ovarian enlargement, delayed bone age, and pituitary hyperplasia, along with a novel finding of a palpable thyroid nodule. Most literature on VWGS focuses on reproductive and systemic effects, with less attention to structural changes in the thyroid. However, biochemical evidence for thyroid dysfunction has been well documented in all previous published cases of VWGS. Structural imaging has usually been limited to diffuse thyroid diseases (such as autoimmune thyroiditis) or glandular enlargement. Diffuse goiter is frequently documented and represents an indication of either autoimmune thyroiditis or iodine deficiency [12]. A cohort of 30 children with VWGS did not routinely perform thyroid ultrasound, and no nodules were described [13]. Other case reports and series have documented diffuse heterogeneous echotexture and increased vascularity consistent with thyroiditis, but without discrete nodular lesions [14]. Consequently, whether the lack of documented nodules in VWGS represents genuine biological rarity, insufficient recognition, or reporting bias remains unclear. This knowledge gap possesses clinical significance because, in the absence of systematic data, clinicians lack evidence-based guidance for managing a child with VWGS who presents with a thyroid nodule. Focal thyroid nodules have not been demonstrated in most of the literature to date (Table 2) [9, 12, 13, 15–33].

**Table 2:** Reported Thyroid Morphological and Structural Abnormalities Documented in Published Pediatric Cases of Van Wyk–Grumbach Syndrome

Year	Author	Study type (N)	Thyroid imaging findings	Thyroid nodule	Ref.
2007	Sanjeevaiah et al	Case report	NR	-	[15]
2012	Baranowski et al	Case report	NR	-	[9]
2012	Patni et al	Case report + review	NR	-	[16]
2015	Bhagwat et al	Case report	NR	-	[17]
2016	Kocova et al	Case series (8)	NR	-	[18]
2018	Leonardi et al	Case report	Enlarged gland with heterogeneous echotexture	-	[19]
2018	Reddy et al	Case report	NR	-	[20]
2020	Lee et al	Case report	Decreased thyroid volume on ultrasound	-	[21]
2020	Gupta et al	Case report	NR	-	[22]
2020	Riaz et al	Case series (10)	Radiologic profile described; thyroid details not reported	-	[23]
2022	Kandasamy et al	Case report	US performed; morphology not detailed	-	[24]
2023	Kusuma Boddu et al	Cohort (30)	Goiter variably reported; structural imaging not detailed	-	[13]
2023	Sherwani et al	Case report	Imaging focused on VWGS; thyroid morphology not detailed	-	[25]
2023	Waddankeri et al	Case series (3)	NR	-	[26]
2023	Marzouk et al	Case report	US and scintigraphy suggest thyroiditis	-	[27]
2024	Nicolescu et al	Case series (2)	Moderate goiter on US in one patient	-	[14]
2024	Rathod et al	Case report	NR	-	[28]
2024	Baioumi et al	Case report	NR	-	[29]
2024	Mukhopadhyay et al	Case series (3)	NR	-	[30]
2025	Ganta et al	Case report	NR	-	[31]
2025	Sivasubramanian et al	Case report	NR	-	[32]
2025	Gil et al	Case report	NR	-	[33]
2026	Cao et al	Case report	NR	-	[12]

“-” indicates that no thyroid nodule was reported. FT4: free thyroxine; NR: not reported; TSH: thyroid-stimulating hormone; US: ultrasound; VWGS: Van Wyk–Grumbach syndrome.

Our case has reassuring sonographic features of a thyroid nodule with low-risk stratification (ACR TI-RADS 3), which favors a benign hyperplastic nodule [15] that showed regression following levothyroxine therapy; however, they do not rule out malignancy, as thyroid nodules in pediatrics carry a higher risk of malignancy than in adults. Although data on the well-documented regression of other TSH-dependent manifestations in VWGS, including ovarian cysts and pituitary hyperplasia [23], are available, the absence of histological confirmation for the thyroid nodule does not definitively establish a causal relationship.

Importantly, future prospective studies of VWGS may evaluate thyroid ultrasound both at diagnosis and after achieving euthyroidism to establish the true prevalence of discrete nodules and their natural history. In the setting of severe hypothyroidism, nodules with low-risk sonographic features may represent TSH-dependent changes that regress with levothyroxine therapy; therefore, careful serial monitoring before invasive evaluation may be a reasonable approach in selected cases.

Recognition of the peripheral nature of precocious puberty in VWGS is essential to avoid unnecessary GnRH agonist therapy. Treatment should instead focus exclusively on thyroid hormone replacement therapy, accompanied by monitoring for the potential development of central precocious puberty [17]. Similarly, pituitary hyperplasia should not prompt surgery or biopsy, as it typically resolves with thyroid hormone replacement alone. A long-term follow-up is required to monitor growth, pubertal progression, thyroid function, and structural changes [12].

This report is limited by its single-patient report, which limits the ability to draw broader conclusions about the occurrence and clinical relevance of thyroid nodules in VWGS. Fine-needle aspiration was not performed because the nodule had low-risk sonographic features (ACR TI-RADS 3) [34] and demonstrated interval regression after levothyroxine therapy. We cannot definitively rule out a coexisting benign neoplasm that regressed coincidentally, although this scenario remains unlikely given the close temporal relationship between TSH reduction and nodule volume regression. In addition, the duration of follow-up was relatively short, preventing assessment of long-term outcomes, including possible recurrence of the thyroid lesion and its impact on growth and pubertal development. Larger studies with extended follow-up are needed to clarify further the relationship between severe hypothyroidism and potentially reversible thyroid nodular changes in this syndrome.

To our knowledge, there are no reports that describe discrete thyroid nodules in patients with VWGS. Thus, the current case report is more of a hypothesis-generating report than a documentation of VWGS. Future case reports are needed to determine whether thyroid nodules occur more frequently in patients with severe, longstanding hypothyroidism than has been reported.

### Learning points

VWGS involved the following diagnostic features in children: isosexual puberty, an advanced bone age, and clinical evidence of longstanding hypothyroidism.

Severe primary hypothyroidism does not simply result in enlarged or diffusely enlarged thyroid but may include thyroid nodular changes.

Elevated TSH levels over time may cause focal thyroid nodular hyperplasia, contributing to thyroid nodules.

Thyroxine replacement therapy may regress not only pituitary hyperplasia and ovarian cysts, but also thyroid nodules, thereby preventing unnecessary invasive surgical procedures.

Thorough clinical evaluation and ultrasound imaging are required when evaluating children with VWGS.

## Data Availability

The data from this report can be obtained from the corresponding author upon request. Confidentiality of patient data will be maintained.
